# Supplementary material for: Functional Characterisation of Banana (Musa spp.) 2-Oxoglutarate-Dependent Dioxygenases Involved in Flavonoid Biosynthesis
Source: Front Plant Sci. 2021 Aug 17;12:701780. doi: 10.3389/fpls.2021.701780 (PMC8415913; doi:10.3389/fpls.2021.701780)
Supplement: Supplementary file 1 [file Data_Sheet_1.docx]

**Supplementary material**

**Table S1: Plant 2-ODDs with proven F3H, FNS I, FLS and ANS functionality, used in phylogenetic analysis.**

| **Name** | **Species** | **GenBank ID** | **Reference** | **Function** |
| --- | --- | --- | --- | --- |
| MtF3H | *Medicago truncatula* | FJ529406.1 | Shen et al. (2010) | F3H |
| OsF3H | *Oryza sativa* | NM_001060692.1 | Kim et al. (2008) | F3H |
| PcF3H | *Petroselinum crispum* | AY230248.1 | Martens et al. (2001) | F3H |
| PhF3H | *Petunia hybrida* | AF022142.1 | Britsch et al. (1986) | F3H |
| ZmF3H | *Zea mays* | AAA91227.1 | Deboo et al. (1995) | F3H |
| AcFLS | *Allium cepa* | KY369209.1 | Park et al. (2017) | FLS |
| AtFLS | *Arabidopsis thaliana* | NP_001190266.1 | Owens et al. (2008) | FLS |
| CuFLS | *Citrus unshiu* | Q9ZWQ9.1 | Wellmann et al. (2002) | FLS |
| FaFLS | *Fragaria x ananassa* | DQ087252 | Almeida et al. (2007) | FLS |
| MdFLS | *Malus domestica* | AY965343 | Halbwirth et al. (2006) | FLS |
| OsFLS | *Oryza sativa* | XP_015624815.1 | Park et al. (2019) | FLS |
| PcFLS | *Petroselinum crispum* | Q7XZQ6.1 | Martens et al. (2003) | FLS |
| PhFLS | *Petunia hybrida* | Q07512.1 | Holton et al. (1993) | FLS |
| ZmFLS | *Zea mays* | BT039956 | Falcone Ferreyra et al. (2010) | FLS |
| AtDMR6 | *Arabidopsis thaliana* | OAO94377.1 | Falcone Ferreyra et al. (2015) | FNSI |
| CjFNSI | *Conocephalum japonicum* | QEP99662.1 | Li et al. (2020) | FNSI |
| CmFNSI | *Conium maculatum* | AY817677 | Gebhardt et al. (2005) | FNSI |
| MeFNSI | *Marchantia emarginata* | QEP99659.1 | Li et al. (2020) | FNSI |
| MpaFNSI | *Marchantia paleacea* | QEP99658.1 | Li et al. (2020) | FNSI |
| PaFNSI | *Plagiochasma appendiculatum* | QEP99657.1 | Li et al. (2020) | FNSI |
| PcFNSI | *Petroselinum crispum* | Q7XZQ8.1 | Martens et al. (2001) | FNSI |
| ZmFNSI | *Zea mays* | NP_001151167.1 | Falcone Ferreyra et al. (2015) | FNSI |
| AcANS | *Allium cepa* | EF192598.1 | Kim et al. (2004) | ANS |
| AtANS | *Arabidopsis thaliana* | NP_194019.1 | Wilmouth et al. (2002) | ANS |
| FaANS | *Fragaria x ananassa* | AY695817.1 | Almeida et al. (2007) | ANS |
| GbANS | *Ginkgo biloba* | ACC66092.1 | Xu et al. (2008) | ANS |
| OsANS | *Oryza sativa* | Y07955.1 | Reddy et al. (2007) | ANS |
| PaANS | *Phytolacca americana* | AB198870.1 | Shimada et al. (2005) | ANS |
| PfANS | *Perilla frutescens* | O04274.1 | Saito et al. (1999) | ANS |
| PhANS | *Petunia hybrida* | AB027454.1 | Nakajima et al. (2001) | ANS |
| SoANS | *Spinacia oleracea* | AB198869.1 | Shimada et al. (2005) | ANS |
| TfANS | *Torenia fournieri* | AB044091.1 | Nakajima et al. (2001) | ANS |
| ZmANS | *Zea mays* | NP_001106074 | Nakajima et al. (2001) | ANS |
| AtGA3ox1 | *Arabidopsis thaliana* | NP_173008.1 | Kawai et al. (2014) | outgroup |

**Table S2: Sequence Read Archive IDs for RNA-Seq raw data used for expression analysis.**

| **biolocial source** | **Sequence Read Archive IDs** |
| --- | --- |
| embryogenic | SRR8761343, SRR8761344, SRR8761345 |
| seedling | SRR7340404, SRR7340406 |
| root | SRR2984591, SRR2984594, SRR2984596, SRR2984598, SRR2984601, SRR4450942, SRR4450934, SRR4450941, SRR4450933, SRR4450944 |
| leaf | SRR6320573, SRR6320439, SRR6320438, SRR6320441, SRR6320440, SRR6320443, SRR5405140, SRR5405141 |
| young_leaf | SRR6320573, SRR6320439 |
| adult_leaf | SRR6320438, SRR6320441 |
| old_leaf | SRR6320440,SRR6320443 |
| pulp | SRR5405133, SRR6320444, SRR5405132,SRR6320447, SRR5405131, SRR6320446, SRR5405130, SRR6320531 |
| pulp S1 | SRR5405133, SRR6320444 |
| pulp S2 | SRR5405132, SRR6320447 |
| pulp S3 | SRR5405131, SRR6320446 |
| pulp S4 | SRR5405130, SRR6320531 |
| peel | SRR5405139, SRR5405138, SRR5405137, SRR6320442, SRR5405136, SRR6320445, SRR5405135, SRR5405134 |
| peel S1 | SRR5405139, SRR5405138 |
| peel S2 | SRR5405137, SRR6320442 |
| peel S3 | SRR5405136, SRR6320445 |
| peel S4 | SRR5405135, SRR5405134 |

**Table S3: Oligonucleotide primers, used in this work.**

| **Name** | **Sequence (5’-3’)** | **Description** |
| --- | --- | --- |
| RS1708 | attB1-ccATGGCTCCCGGCACCGCAGTCCTTCCC | fwd Primer with attB1 for *Musa*F3H1 (Ma02_t04650) CDS |
| RS1709 | *attB2-*aAGCCAAGATCTCATCGAGGCCTGTAGCC | rev Primer with attB2 for *Musa*F3H1 (Ma02_t04650) CDS |
| RS1710 | *attB1-*ccATGGCCCCCGGCACTGCATTGCTTCCC | fwd Primer with attB1 for *Musa*F3H2 (Ma07_t17200) CDS |
| RS1712 | *attB2-*aAGCTAGGATCTCATTTAGGCATTTCGCC | rev Primer with attB2 for *Musa*F3H2 (Ma07_t17200) CDS |
| RS1681 | *attB1-*ccATGGAGGTGGAGAGGGTGCAGGCC | fwd Primer with attB1 for *Musa*FLS1 (Ma03_t06970) CDS |
| RS1682 | attB2-aCTGCGGAAGCTTGTTCAGCTTGCAG | rev Primer with attB2 for *Musa*FLS1 (Ma03_t06970) CDS |
| RS1695 | attB1-ccATGGAGGTGGAGAGGGTTCAAGTCATCGCG | fwd Primer with attB1 for *Musa*FLS3 (Ma10_t25110) CDS |
| RS1696 | attB2-aTTGCGGAAGCTTGTTGAGCTTGCAGTAGGCG | rev Primer with attB2 for *Musa*FLS3 (Ma10_t25110) CDS |
| RS1704 | attB1-ccATGGCCACCAAGGTCGTGTCTG | fwd Primer with attB1 for *Musa*ANS (Ma05_t03850) CDS |
| RS1705 | attB2-aGTTTGGGGTCTTGAAGTCCCCCCG | rev Primer with attB2 for *Musa*ANS (Ma05_t03850) CDS |
| RS2101 | attB1-ccATGGCTCCTACAACAATAAC | fwd Primer with attB1 for *PcFNSI* CDS |
| RS2102 | attB2-AGCTAAATTTTCATCTGCAC | rev Primer with attB2 for *PcFNSI* CDS |

attB1: GGGGACAAGTTTGTACAAAAAAGCAGGCT, attB2: GGGGACCACTTTGTACAAGAAAGCTGGGT

**File S1: CDS of *Musa2-*ODDs from this work.**

Variant positions to the Pahang reference genome are given in bold-underlined.

*Musa*F3H1

ATGGCTCCCGGCACCGCAGTCCTTCCCACCAACGAGCAGACCTTGAGGGCGAGCTTCGTCCGGGACGAGGACGAGCGTCCCAAGGTGGCTTACAACCAGTTCAGCCTTGACATCCCCGTGATCTCGCTCTCCGGGATTGATGACGACGCCGCGGGCGGGAAGAGGGCGGAGATCCGCCGCAAGGTCGTGGAGGCCTGCGAGGACTGGGGTATATTCCAGGTGGTGGACCACGGGGT**G**GACGCCGGCCTTATCTCCGACATGACTAGGCTGGCCAAAGAGTTCTTCGCGCTGTCCCCGGA**T**GAGAAGCTCTTGTTCGACATGTCCGGTGGGAAGAAGGGAGGGTTCATCGTCTCCAGCCACCTCCAGGGCGAGGCAGTTCAGGACTGGAGAGAGATCGTGACGTACTTCTCATACCCAATTCGGGCTCGGGACTACTCGCG**G**TGGCCGGACAAGCCGGAAGGGTGGAGATCCGTGGTGGAGTCGTACAGCGAGAAGCT**G**ATGGGCTTGGCGTGCAAGCTCTTGGAGGTGTTGTCTGAGGCTATGGGGCTCGACAAGGAGGCCCTCACCGAGGCCTGCATCGACATGGATCAGAAGGT**G**GTCGTCAACTACTACCCCAAGTGCCCCCAGCCGGACCTCACTCTTGGCCTCAAACGACACACCGATCCCGGCACCATCACCCTCCTCCTCCAAGACCAAGTCGGCGGCCTCCAAGCCACCAAGGATGGCGGCAAGACATGGATCACGGTTCAGCCTGTGGAGGGAGCTTTTGTGGTCAACCTCGGGGACCATGGCCATTTTCTGAGCAATGGGAGGTTCAAGAATGC**A**GACCACCAGGCTGTGGTGAACTCAAACT**G**CAGCAGGCTCTCCATCGCCACGTTTCAGAACCCCGCGCCGGAGGCAATCGTGTATCCGCTGGCAGTAAGGGAGGGAGAGAAGCCGATTCTGGA**C**GAGCCCATCACGTTTGCCGAGATGTACCGCAGGAAGATGAGCCGAGACCTCGAGCTCGCCAAGCTCAAGAAACTGGCCAAGACGGAGCAG**C**AGCAG**A**AGCCGGAGCTACT**A**GAGAAGACTAAGGACATCAACTTGGCCAAGGCTACAGGCCTCGATGAGATCTTGGCT

*Musa*F3H2

ATGGCCCCCGGCACTGCATTGCTTCCCACGAACGAGGCGACCCTGAGGGCGAGCTTCGTGCGGGACGAGGACGAGCGACCCAAGGTGGCCTA**C**AACCA**G**TTCAGCAGCGACATCCC**C**GTGATCTCGCTCGCCGGGGTTGACGACGAGGACGGGGCCACCGGCGGGCGGAGGGCGGAGATCCGCCGCAAGATCGTGGA**A**GCCTGCGAGGACTGGGGCTTATTCCAGGTG**T**TGGACCATGGAGTGGACGCCGGCATGATCTCCGAGATGACCCGGCTCGCCCGGGATTTCTTCGCGCTACCCCCCGAGGAGAAGCT**A**CGGTTCGACATGTCCGGCGGAAAAAAGGGAGGCTTCATCGTGTCCAGCCATCTCCAGGGTGAGGCAGTGCAGGACTGGCGAGAGATCGTGACTTACTTCTCGTACCCGATCGGAGCTCGGGACTACTCGAGGTGGCCGGATAAGCCAGAAGGGTGGAGGTCGGTGGTGGAGTCATACAGCGAGAAGCTGATGGGGTTGGCGTGCAAGCTGCTGGAGGTGTTGTCGGAGGCAATGGGGCTCGACAAGGAAGCCCTCAC**T**GATGCCTGCGTAGACATGGATCAGAAGGTGGTGGTCAACTACTACCCCA**T**GTGCCCCCAGCCCGACCTCACCCTCGGCCTCAAACGCCACACCGATCCCGG**C**ACCATCACCCTCCTCCTCCAAGACCAAGTCGGCGGACTCCAAGCCACCAAGGACGGCGGCAAGACGTGGATCACAGTTCAGCCCGTGGAGGGAGCTTTCGT**T**GTCAACCTCGGGGACCATGGCCATTATCTGAGCAACGGGAGGTTCAAGAACGC**C**GATCATCAGGCGGTGGTGAACTCGAGCTGCAGCAGGCTGTCGATCGCCACGTTTCAGAACCCAGCGCCGGAGGCGATCGTGTATCCCCTGGCGATAAGGGAGGGAGA**G**AAGCCGATCCTGGACGAGCCCATCACGTTCAGCGAGATGTACCGTAGGAAGATGAGCCGAGATCTC**A**AGCTTGCCAACCT**T**AAGAA**G**CTGGCCA**A**GGCGGAGCATCAGCGGC

*Musa*FLS1

ATGGAGGTGGAGAGGGTGCAGGCCATCGCGTCCCTGAGCGTGGCCACCAACGACATACCGCCGGAGTTCGTGAGGTCGGAGCACGAGCAGCCGGGTATCACCACGTACCGCGGCC**T**GGTCCCGGAGATCCCGGTGATCGACCTCGAAGACGGGGACGAAGGCCGGGTGACGCGCGCCATCGCGGAGGCCAGCCAGGAGTGGGGCATCTTCCAGCTGGTGAACCACGGCATCCCCGGGGAGGTGATCCGGGCGCTGCAGCGCGTGGGCAGGGAGTTCTTCGAGCTGCCACCGGAGGAGAAGGAGAAGTACGCGGCGGCGCCGGGGAGCCTCCAGGGCTACGGAACCAAGCTGCAGAAGGACTTGGAAGGCAAGAAGGCGTGGGTGGACTTCCTCTTCCACAACATCTGGCCGCCGACGCACGTCGACCACCGCGCATGGCCGGAGAATCC**G**GTGGATTACAGGAAGGCAAATGAGGAGTACGCCAAACATTTGGTGGGATTGGTGGAGAAGATGTTGGTAAGCCTGTCCAAGGGACTGGGGCTGGAGGCCGACGTCCTCAAGCACGCAGTGGGAGGGGACGACTTGGAGTTCCTCCTCAAGATCAACTACTACCCGCCGTGCCCGAGACCCGACCTCGCCCTCGGCGTGGTGGCTCACACCGACATGTCCGCCATCACCATCCTGATCCCCAACGACGTCCCCGGCCTCCAGGTCTTCAAGGACGACCACTGGTTCGACGCCAAGTACGTCCCCGACGCCATCATCGTCCACATCGGGGACCAGATCGAGAAACTGAGCAACGGCAGGTACAAGAGCGTGCTGCACCGGACGACGGTGAACAAGGAGAAGGCGAGGATGTCGTGGCCTGTGTTCTGCTCCCCGCCGGGCGAGACGGTCATTGGGCCTCTGCCGCAGCTCGTCAGCGACGAACAGCCCGCTCAGTACAAGACGAAGAAGTACAAGGACTATGCTTTCTGCAAGCTGAACAAGCTTCCGCAG

*Musa*FLS3

ATGGAGGTGGAGAGGGTTCAAGTCATCGCTTCCGTCTGCGCCGCCGACGGTGTTATGCCTCCGGAGTTCATACGGTCGGAGCACGAGCAGCCGGGCATCACCACCTACCGCGGCCCCGCGCCGGAGATCCCCGTCATCGACCTCGCGGGCGCCGACCGGGA**C**CGGTTGACGATCGCCGTCGCCGAGGCCAGCCGGGAGTGGGGAATCTTCCAACTGCTGAACCACGGGATCCCGAGGGAGGTGATCCGTGAGCT**T**CAGCGCGTCGGCAAG**C**AATTCTTCGAGTTGCCGCAGGAGGAGAAGGAGATGTACGCGATGGAAT**G**CAAACCGGGGAGCTCGGAAGGATATGGGAC**C**AAGCTGCAGAGGGAGTTGGAGGGCAAAAAGGC**T**TGGGT**A**GACTTCTTCTTCCACTAC**A**T**T**TCGCCGCCGGCTCGCGTCAACCACGCCATCTGGCCCAAGAACCCT**G**CTGATTA**T**AGGAAAGCAAACGAGGAATATG**G**CAAACACCTGGTGGG**T**CTGGTGGACAAGATGCTGA**T**GAC**G**CTGTCGAGGGGACTGGGACTGGAGGAGCATGTCCTCAAGGGAGCACTCGGTGGAGATGGACTGGAGCTACG**T**CTAAAGATGAACTACTACCCACCGTGTCCTCGGCCTGACCTGGCTCTCGGCGTGGTGGCGCACACTGACATGTGTGCTATCACCTTCCTCGTCCCTAACCTTGTGCCGGGTTTGCAGGTCTTCAAAGATGAGCACTGGATTGACGTCAACTTCATTCCTAATGCTGTCATCGTCCACATCGGTGATCAGATCGAGATTTTGAGCAATGGAACATACAAGAGCGTGCTGCACAGAACGACCGTGAACAAAGAGAAGGTGAGGATGTCTTGGCCAGTGTTCTGCGCGCCTCCTGGTGAGATGGTAATTGGCCCTCTGCAACAGCTCGTCGGCGATGAGAGCCCAGCCAAGTATAAGCCGAAGAAGTACAAAGACTACGCCTACTGCAAGCTCAACAAGCTTCCGCAA

*Musa*ANS

ATGGCCACCAAGGTCGTGTCTGTGGCGCCCAGGGTGGAGATCCT**G**GC**G**AAGAGCGGCATCAACGAGATCCCGACCGAGTACGTCCGCCCCGAGTCGGAGCGGCTCGACCTCGGTGACGCGTTCGAGGAGGTGAAGAAGGCGGCGGAGGGGCCTCAGATTCCTGTGGTGGACCTCCAGGGTTTCGACTCGCCTGATGAAGAGGTGAGAAGGGCGTGCGTGGAGGAGGTGAGGAAGGCGGCGACGGAGTGGGGGGTGATGCACATCGTGAATCACGGCATCCCGTTGGAGCTCGTCGAGCAGCTGAGGAAGGTGGGGAAGGAGTTCTTCGACCTGCCCATAGAGCAGAAGGAGCAGTACGC**A**AACGACCAGTCATCAGGGAAGATCCAAGGGTACGGGAGCAAGCTGGCGAACAACGCGAGTGGGCAGCTCGAGTGGGAGGACTACTTCTTCCACCTTATATTCCCGGAGGAGAAGATCAACATCTCCATTTGGCCCAAGCAACCAACCGACTACATTGAGGTGACGAAGAAGTTCGGGAGGCAGCTGAGGGCGGTGGTCACCAAGATGCTGGAAGTTCTTTCCCTGGGTCTTGGACTGGAAGAGGGGAAGCTGGACAGGGAACTCGGAGGGATGGAGGACCTGCTGATGCAGTTGAAGATCAACTACTACCCTATCTGCCCACAGCCCGATCTCGCCCTCGGCGTCGAGGCGCACACCGACATCAGCGCGCTCTCCTTCATCCTCCACAACATGGTGCCGGGGCTGCAGATCTACTACGGCGGCAGGTGGGTCACCGCCAAATG**T**GTGCCGGACTCCATCATCATGCACGTCGGAGACTGCCTCGAGATCCTGAGCAATGGGCAGTACAAGAGCATCCTCCACCGCGGGCTCGTCAACAAGGAGAAGGTGCGCATCTCCTGGGCGGTCTTCTGCGAGCCTCCCAAAGACAAGATCGTGCTGAAGCCACTGGAGGAGCTCGTGGCCGACGGGACGCCGGCCAAGTTCCCCCCGCGCACCTTCGAGCAGCACATCCAGCACAAGCTCTTCAAGAAGACTCGGGGGGACTTCAAGACCCCAAAC

**File S2: Multiple sequence alignment of flavonoid biosynthesis related 2-ODDs from banana and plant proteins with demonstrated 2-ODD activity.**

Substrate binding residues are highlighted in grey. Bold-underlined amino acids are involved in binding ferrous iron or 2-oxoglutarate (Chua et al., 2008). Asterisks mark conserved residues, colons indicate very similar amino acids and full stops indicate weakly similar properties.

AtF3H -------MAP-----GTLTELA---GESKLNSKFVRDEDERPKVAYNV----------FS 35

MusaF3H1 -------MAP-----GTAVLPT---NEQTLRASFVRDEDERPKVAYNQ----------FS 35

MusaF3H2 -------MAP-----GTALLPT---NEATLRASFVRDEDERPKVAYNQ----------FS 35

OsF3H -------MAPV----ATTFLPTAS-NEATLRPSFVRDEDERPRVAYNQ----------FS 38

ZmF3H -------MAPVSIS-AVPFLPTAAEGETNVRASFVREEDERPKVPHDR----------FS 42

AcFLS --------MEVE---RVQAIATLTANLGTIPSEFIRSDHERPDLTTYH---------GPV 40

MusaFLS1 --------MEVE---RVQAIASLSVATNDIPPEFVRSEHEQPGITTYR---------GLV 40

MusaFLS3 --------MEVE---RVQVIASVCAADGVMPPEFIRSEHEQPGITTYR---------GPA 40

MusaFLS4 --------MEVE---RVQVIASVCAADGVMPPEFIRSEHEQPGITTYR---------GPA 40

MusaFLS2 --------MAQV---KVQAIASMLHSKDTIPPEFVRVEDEQPGATTYR---------GPA 40

AtFLS1 --------MEVE---RVQDISSSSLLTEAIPLEFIRSEKEQPAITTFR---------GPT 40

ZmFLS --------MGGETHLSVQELAAS---LGALPPEFVRSEQDQPGATTYRG--------AAV 41

OsFLS -------MAEVQ---SVQALASS---LAALPPEFVRSEHERPGATTFRG--------GDA 39

AtANS -------MVAVE---RVESLAKS--GIISIPKEYIRPKEELESINDVFLEEKK----EDG 44

MusaANS MATK--VVSVAP---RVEILAKS--GINEIPTEYVRPESERLDLGDAFEEVKKA---AEG 50

ZmANS MESSPLLQLPAA---RVEALSLS—GLSAIPPEYVRPADERAGLGDAFDLARTHANDHTA 55

OsANS -------MTDAE--LRVEALSLS—GASAIPPEYVRPEEERADLGDALELARAASDDDAT 49

. : .::* :

AtF3H DEIPVISLAGI--DDVD----GKRGEICRQIVEACENWGIFQVVDHGVDTNLVADMTRLA 89

MusaF3H1 LDIPVISLSGI--DDD--AAGGKRAEIRRKVVEACEDWGIFQVVDHGVDAGLISDMTRLA 91

MusaF3H2 SDIPVISLAGV--DDEDGATGGRRAEIRRKIVEACEDWGLFQVLDHGVDAGMISEMTRLA 93

OsF3H DAVPVISLQGI--DE------AARAEIRARVAGACEEWGIFQVVDHGVDAGLVADMARLA 90

ZmF3H DEVPVVSLEGI--DGP-----RRRAEIRGRVAAACEDWGIFQVVDHGVDAALVADMARLA 95

AcFLS PELPVIDLANS-----------SQENVVKQISEAAREYGIFQLVNHGIPNEVINELQRVG 89

MusaFLS1 PEIPVIDLEDG-----------DEGRVTRAIAEASQEWGIFQLVNHGIPGEVIRALQRVG 89

MusaFLS3 PEIPVIDLAGA-----------DRDRLTIAVAEASREWGIFQLLNHGIPREVIRELQRVG 89

MusaFLS4 PEIPVIDLAGA-----------DRDRLTIAVAEASREWGIFQLLNHGIPREVIRELQRVG 89

MusaFLS2 PAVPVIDLADT-------------DRVVDAIADASREWGIFQLVNHGIPAAVIGELQRVG 87

AtFLS1 PAIPVVDLSDP-----------DEESVRRAVVKASEEWGLFQVVNHGIPTELIRRLQDVG 89

ZmFLS PDAPVIDISEP--------------GFGARMAAAAREWGLFQVVNHGVPSAAVAELQRVG 87

OsFLS PEIPVIDMAAP--------------ESAARVAEAAAEWGLFQVVNHGVPAAAVAELQRVG 85

AtANS PQVPTIDLKNI--ESDD---EKIRENCIEELKKASLDWGVMHLINHGIPADLMERVKKAG 99

MusaANS PQIPVVDLQGF--DSPD---EEVRRACVEEVRKAATEWGVMHIVNHGIPLELVEQLRKVG 105

ZmANS PRIPVVDISPFLDSSSQ---QQQRDECVEAVRAAAADWGVMHIAGHGIPAELMDRLRAAG 112

OsANS ARIPVVDISAF---DND---GDGRHACVEAVRAAAEEWGVMHIAGHGLPGDVLGRLRAAG 103

*.:.: : *. ::*:::: .**: : : .

AtF3H RDFFALPPEDKLRF--DMSGGKKGGFIVSSHLQGEAVQDWREIVTYFSYPVRNRDYSRWP 147

MusaF3H1 KEFFALSPDEKLLF--DMSGGKKGGFIVSSHLQGEAVQDWREIVTYFSYPIRARDYSRWP 149

MusaF3H2 RDFFALPPEEKLRF--DMSGGKKGGFIVSSHLQGEAVQDWREIVTYFSYPIGARDYSRWP 151

OsF3H RDFFALPPEDKLRF--DMSGGKKGGFIVSSHLQGEAVKDWREIVTYFSYPVKSRDYSRWP 148

ZmF3H RDFFALPPEDKLRF--DMSGGKKGGFIVSSHLQGEAVQDWREIVTYFSYPVKARDYSRWP 153

AcFLS KEFFQLPQEEKEVYATVPDSGSFEGYGTKLQKDLEGKKAWVDYLFHNVWPKHKINYKFWP 149

MusaFLS1 REFFELPPEEKEKYAAAP--GSLQGYGTKLQKDLEGKKAWVDFLFHNIWPPTHVDHRAWP 147

MusaFLS3 KQFFELPQEEKEMYAMECKPGSSEGYGTKLQRELEGKKAWVDFFFHYISPPARVNHAIWP 149

MusaFLS4 KEFFELPQEEKEMYAMEFKPGSSEGYGTKLQRELEGKKAWVDFFFHYVSPPARVNHAIWP 149

MusaFLS2 REFFELPQEEKESYAADPRSGSIEGYGTQIQKDPNGKKAWGDYLFHNVWPPSRINHGMWP 147

AtFLS1 RKFFELPSSEKESVAKPEDSKDIEGYGTKLQKDPEGKKAWVDHLFHRIWPPSCVNYRFWP 149

ZmFLS RAFFALPTEEKERYAMDPASGKIEGYGTKLQRDLEGKKTWNDFFFHVVAPPEKVDHAVWP 147

OsFLS REFFALPQEEKARYAMDASSGKMEGYGSKLQKDLEGKKAWADFFFHNVAPPAMVNHDIWP 145

AtANS EEFFSLSVEEKEKYANDQATGKIQGYGSKLANNASGQLEWEDYFFHLAYPEEKRDLSIWP 159

MusaANS KEFFDLPIEQKEQYANDQSSGKIQGYGSKLANNASGQLEWEDYFFHLIFPEEKINISIWP 165

ZmANS TAFFALPVQDKEAYANDPAAGRLQGYGSRLATNTCGQREWEDYLFHLVHPDGLADHALWP 172

OsANS EAFFALPIAEKEAYANDPAAGRLQGYGSKLAANASGKREWEDYLFHLVHPDHLADHSLWP 163

** *. :* *: : . * : . : * : **

AtF3H DKPEGWVKVTEEYSERLMSLACKLLEVLSEAM-GLEK-ESLTNAC----------VDMDQ 195

MusaF3H1 DKPEGWRSVVESYSEKLMGLACKLLEVLSEAM-GLDK-EALTEAC----------IDMDQ 197

MusaF3H2 DKPEGWRSVVESYSEKLMGLACKLLEVLSEAM-GLDK-EALTDAC----------VDMDQ 199

OsF3H DKPAGWRAVVEQYSERLMGLACKLLGVLSEAM-GLDT-NALADAC----------VDMDQ 196

ZmF3H DKPAAWRAVVERYSEQLMALACRLLGVLSEAM-GLDT-EALARAC----------VDMDQ 201

AcFLS RNPPAYRKANEEYTKHLQVVVDKMHSYLSLGL-GLES-HVLKEAVGG--------DDLEY 199

MusaFLS1 ENPVDYRKANEEYAKHLVGLVEKMLVSLSKGL-GLEA-DVLKHAVGG--------DDLEF 197

MusaFLS3 KNPADYRKANEEYGKHLVGLVDKMLMTLSRGL-GLEE-HVLKGALGG--------DGLEL 199

MusaFLS4 KNPSDYRKANEEYAKHLVGLVDKMLTTLSRGL-GLEE-HVLKGALGG--------DGLEL 199

MusaFLS2 RQPSSYREANEEYTKYLLGILDKILDSLSLGL-GLEG-SALKKALGG--------EEMDM 197

AtFLS1 KNPPEYREVNEEYAVHVKKLSETLLGILSDGL-GLKR-DALKEGLGG--------EMAEY 199

ZmFLS RSLAGYREANEEYCRHMQRLTRELFEHLSLGL-GLHG-GAMAEAFGG--------DGLVF 197

OsFLS SHPAGYREANEEYCKHMQRLARKLFEHLSTAL-GLDG-GAMWEAFGG--------DELVF 195

AtANS KTPSDYIEATSEYAKCLRLLATKVFKALSVGL-GLEP-DRLEKEVGGL-------EELLL 210

MusaANS KQPTDYIEVTKKFGRQLRAVVTKMLEVLSLGL-GLEE-GKLDRELGGM-------EDLLM 216

ZmANS AYPPDYIAATRDFGRRTRDLASTLLAILSMGLLGTDRGDALEKALTTTTTRTAADDDLLL 232

OsANS ANPPEYVPVSRDFGGRVRTLASKLLAILSLGL-GLPE-ETLERRLRGHEL-AGVDDDLLL 220

: . : : : ** .: * :

AtF3H KIVVNYYPKCPQPDLTLGLKR**H**T**D**PGTITLLLQDQVGGLQATRDNGKTWITVQPVEGAFV 255

MusaF3H1 KVVVNYYPKCPQPDLTLGLKR**H**T**D**PGTITLLLQDQVGGLQATKDGGKTWITVQPVEGAFV 257

MusaF3H2 KVVVNYYPMCPQPDLTLGLKR**H**T**D**PGTITLLLQDQVGGLQATKDGGKTWITVQPVEGAFV 259

OsF3H KVVVNFYPKCPQPDLTLGLKR**H**T**D**PGTITLLLQDLVGGLQATRDAGKTWITVQPIPGSFV 256

ZmF3H KVVVNFYPRCPQPDLTLGLKR**H**T**D**PGTITLLLQDLVGGLQATRDGGRTWITVQPVEGAFV 261

AcFLS LLKINYYPPCPRPNLALGVVA**H**T**D**MSSLTILVPNEVPGLQVFKDD--HWFDAKYIPNALI 257

MusaFLS1 LLKINYYPPCPRPDLALGVVA**H**T**D**MSAITILIPNDVPGLQVFKDD--HWFDAKYVPDAII 255

MusaFLS3 RLKMNYYPPCPRPDLALGVVA**H**T**D**MCAITFLVPNLVPGLQVFKDE--HWIDVNFIPNAVI 257

MusaFLS4 RLKMNYYPPCPRPDLALGVVA**H**T**D**MCAITFLVPNLVPGLQVFKDE--HWIDVNFIPNAVI 257

MusaFLS2 LLKINYYPPCPRPDLALGVVA**H**S**D**LSAVTILVPSDVPGLQISKDD--RWIDIDYVPGALI 255

AtFLS1 MMKINYYPPCPRPDLALGVPA**H**T**D**LSGITLLVPNEVPGLQVFKDD--HWFDAEYIPSAVI 257

ZmFLS LQKINFYPPCPQPELTLGVAP**H**T**D**MSTLTVLVPNEVQGLQVFKDG--QWYEAKYVPDALI 255

OsFLS LHKINFYPPCPEPELTLGVAP**H**T**D**MSTFTVLVPNDVQGLQVFKDG--HWYDVKYVPDALI 253

AtANS QMKINYYPKCPQPELALGVEA**H**T**D**VSALTFILHNMVPGLQLFYEG--KWVTAKCVPDSIV 286

MusaANS QLKINYYPICPQPDLALGVEA**H**T**D**ISALSFILHNMVPGLQIYYGG--RWVTAKCVPDSII 274

ZmANS QLKINYYPRCPQPELAVGVEA**H**T**D**VSALSFILHNGVPGLQVLHGA--RWVTARHEPGTII 290

OsANS QLKINYYPRCPRPDLAVGVEA**H**T**D**VSALSFILHNGVPGLQVHHAG--SWVTARPEPGTIV 278

:*:** **.*:*::*: *:* .:.:: . * *** * .:.:

AtF3H VNLGDHGH-----------FLSNGRFKNAD**H**QAVVNSNSS**R**L**S**IATFQNPAPDAT-VYPL 303

MusaF3H1 VNLGDHGH-----------FLSNGRFKNAD**H**QAVVNSNCS**R**L**S**IATFQNPAPEAI-VYPL 305

MusaF3H2 VNLGDHGH-----------YLSNGRFKNAD**H**QAVVNSSCS**R**L**S**IATFQNPAPEAI-VYPL 307

OsF3H VNLGDHAHIMHLLGNVNLQYLSNGRFKNAD**H**QAVVNSDCC**R**L**S**IATFQNPAPDAM-VYPL 315

ZmF3H VNLGDHGH-----------LLSNGRFKNAD**H**QAVVNSECS**R**L**S**IATFQNPAPDAT-VYPL 309

AcFLS CHIGDQLE-----------ILSNGKYKSVL**H**RTTVNKEKS**R**M**S**WPVFCSPPGDTM-IGPL 305

MusaFLS1 VHIGDQIE-----------KLSNGRYKSVL**H**RTTVNKEKA**R**M**S**WPVFCSPPGETV-IGPL 303

MusaFLS3 VHIGDQIE-----------ILSNGTYKSVL**H**RTTVNKEKV**R**M**S**WPVFCAPPGEMV-IGPL 305

MusaFLS4 VHIGDQIE-----------ILSNGTYKSVL**H**RTTVNKEKV**R**M**S**WPVFCAPPGEMV-IGPL 305

MusaFLS2 IHIGDQIE-----------ILSNGKYKSVL**H**RATVNKEKA**R**I**S**WPVFCSPPPEMT-VGPL 303

AtFLS1 VHIGDQIL-----------RLSNGRYKNVL**H**RTTVDKEKT**R**M**S**WPVFLEPPREKI-VGPL 305

ZmFLS VHIGDQIE-----------IFSNGAYKAVL**H**RTTVNKEKT**R**M**S**WPMFVEPPGELV-VGPH 303

OsFLS IHIGDQIE-----------ILSNGRYKAVL**H**RTTVDKDRT**R**M**S**WPVFVEPPPEHV-VGPH 301

AtANS MHIGDTLE-----------ILSNGKYKSIL**H**RGLVNKEKV**R**I**S**WAVFCEPPKDKIVLKPL 317

MusaANS MHVGDCLE-----------ILSNGQYKSIL**H**RGLVNKEKV**R**I**S**WAVFCEPPKDKIVLKPL 323

ZmANS VHVGDALE-----------ILSNGRYTSVL**H**RGLVNREAV**R**I**S**WVVFCEPPPDSVLLHPL 339

OsANS VHVGDALE-----------ILTNGRYTSVL**H**RGLVSRDAV**R**L**S**WVVFCEPPPESVLLQPV 327

::** ::** :. *: *. . *:* * *. : : *

AtF3H K--VREGEKAILEEPITFAEMYKRKMGRDLELARLKKLAKEERDHK-EVDKP-------- 352

MusaF3H1 A--VREGEKPILDEPITFAEMYRRKMSRDLELAKLKKLAKTEQQQKPELLEK-------T 356

MusaF3H2 A--IREGEKPILDEPITFSEMYRRKMSRDLKLANLKKLAKAEHQRL-ELPEN-------A 357

OsF3H A--VRDGEEPILEEPITFAEMYRRKMARDLELAKLKKKAKEQRQLQ-QAALPPPPPTQVA 372

ZmF3H A--VRDGEAPILDHPITFAEMYRRKMARDIELARLKKQAKADKKQQ-QQSAN-------- 358

AcFLS PQLVNDEN-PPKFKTKKYKDYAYCKINK---------LPQ-------------------- 335

MusaFLS1 PQLVSDEQ-PAQYKTKKYKDYAFCKLNK---------LPQ-------------------- 333

MusaFLS3 QQLVGDES-PAKYKPKKYKDYAYCKLNK---------LPQ-------------------- 335

MusaFLS4 QQLVGDES-PAKYKPKKYKDYAYCKLNK---------LPQ-------------------- 335

MusaFLS2 PQFVSDQN-PAKYKTKKYKDYQYCKLNK---------LPQ-------------------- 333

AtFLS1 PELTGDDN-PPKFKPFAFKDYSYRKLNK---------LPLD------------------- 336

ZmFLS PKLVTEES-PAKYKAKKYKDYQHCKINK---------LPM-------------------- 333

OsFLS PQLVTDGS-PAKYKAKKFKDYRHCKINK---------LPM-------------------- 331

AtANS PEMVSVES-PAKFPPRTFAQHIEHKLFG---------KEQEELVSE-KN----------- 355

MusaANS EELVADGT-PAKFPPRTFEQHIQHKLFK---------KTRGDFKTP-------------- 359

ZmANS PELVTEGH-PARFTPRTFKQHLDRKLFK---------KKQQHKAKA-EEEDGG------N 382

OsANS QELLADGAGKPLFAPRTFKQHVQRKLFKKL-------KDQQDNNAA-AASNG-------- 371

. : : *:

AtF3H ----------VDQIFA 358

MusaF3H1 KDINLAKATGLDEILA 372

MusaF3H2 NDINVAKAKCLNEILA 373

OsF3H AELAAQKPKSLDEILA 388

ZmF3H KEFADSKP--LDAIFA 372

AcFLS ---------------- 335

MusaFLS1 ---------------- 333

MusaFLS3 ---------------- 335

MusaFLS4 ---------------- 335

MusaFLS2 ---------------- 333

AtFLS1 ---------------- 336

ZmFLS ---------------- 333

OsFLS ---------------- 331

AtANS ---------------D 356

MusaANS ---------------N 360

ZmANS GDHHRHEP---PPQTN 395

OsANS ------------MITK 375

**
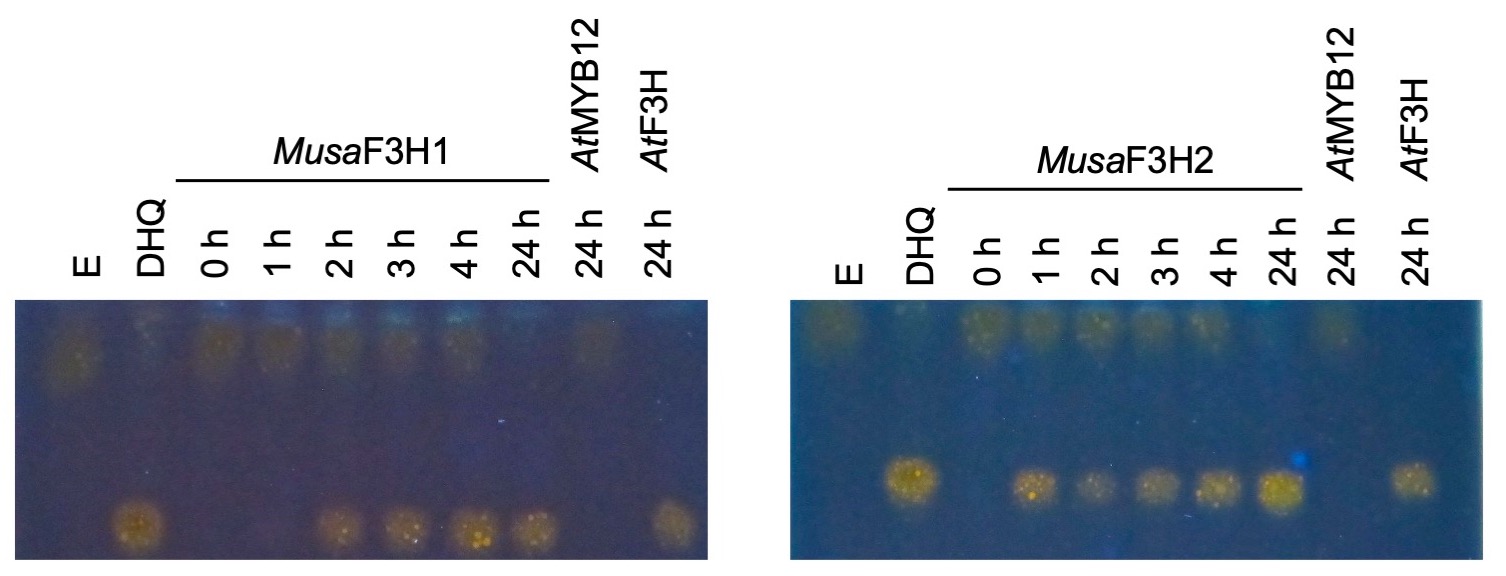
**

**File S3: *Musa*F3H1 and *Musa*F3H2 convert eriodictyol to dihydroquercetin.**

HPTLC analysis of F3H bioconversion assays using extracts from *E. coli* expressing recombinant *Musa*F3H1 or *Musa*F3H2. The substrate eriodictyol (E) and the product dihydroquercetin (DHQ) were used as standards. *At*F3H was used as a positive control. *At*MYB12 was used as a negative control.


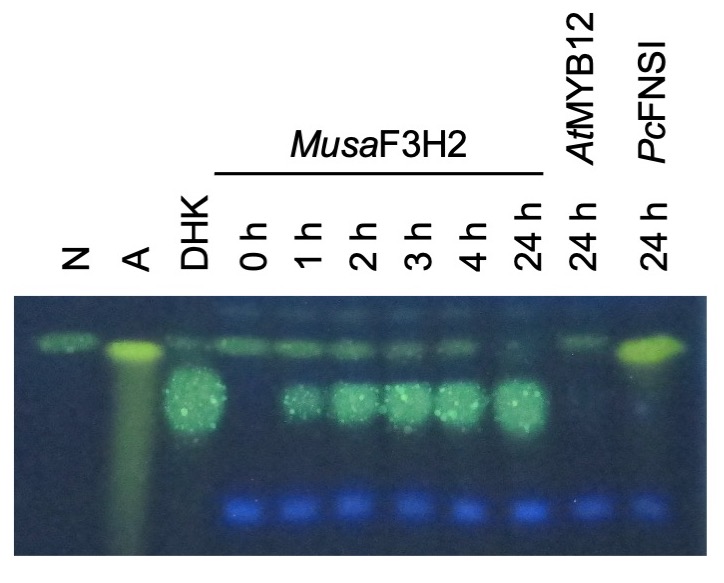


**File S4: Analysis of FNSI side activity of *Musa*F3H2.**

HPTLC analysis of FNSI bioconversion assay using extracts from *E. coli* expressing recombinant *Musa*F3H2. The substrate naringenin (N) and the product apigenin (A), as well as the F3H product dihydrokaempferol (DHK) were used as standards. *Pc*FNSI was used as a positive control. *At*MYB12 was used as a negative control.

**
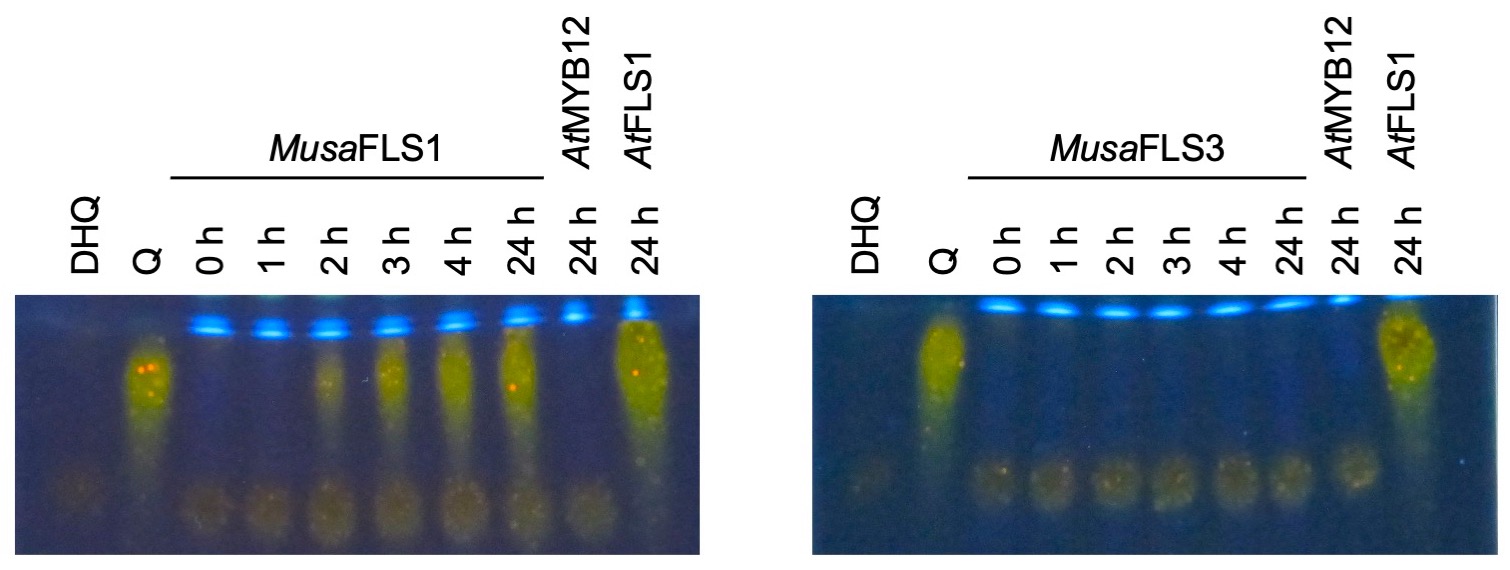
**

**File S5: MusaFLS1 converts dihydroquercetin to quercetin.**

HPTLC analysis of a FLS bioconversion assays using extracts from *E. coli* expressing recombinant *Musa*FLS1 or *Musa*FLS3. The substrate dihydroquercetin (DHQ) and the product quercetin (Q) were used as standards. *At*FLS1 served as a positive control and *At*MYB12 was used as a negative control.

**
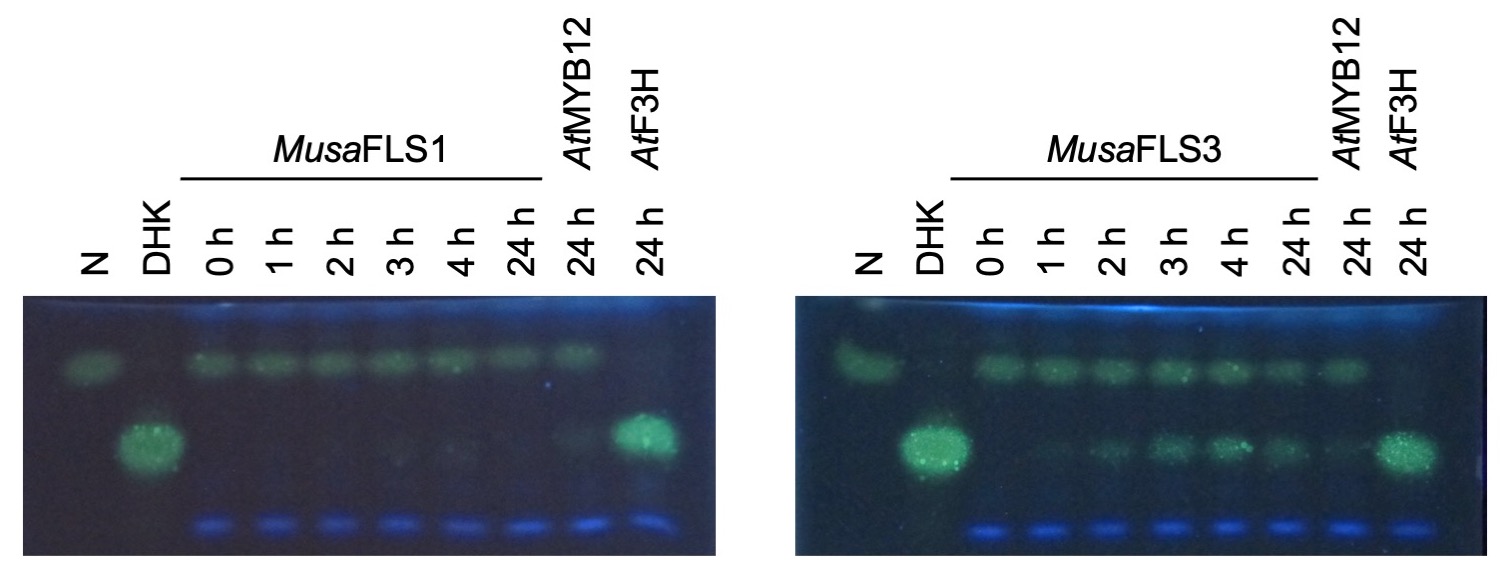
**

**File S6: Analysis of F3H side activity of *Musa*FLS1 and *Musa*FLS3.**

HPTLC analysis of F3H bioconversion assay using extracts from *E. coli* expressing recombinant *Musa*FLS1 or *Musa*FLS3. The substrate naringenin (N) and the product dihydrokaempferol (DHK) were used as standards. *At*F3H was used as a positive control. *At*MYB12 was used as a negative control.

**File S7: Analysis of recombinant *Musa*FLS1 and *Musa*FLS3 in *E. coli* by SDS-PAGE.**

Sample separation was performed by SDS-PAGE (Laemmli, 1970) using a 4 % stacking gel and 12 % resolving gel. Protein staining was performed using colloidal Coomassie Brilliant Blue G-250 (Dyballa and Metzger, 2012). The first lanes for each sample show the supernatant of *E. coli* cultures without addition of inducing arabinose, the second ones show the supernatant of induced *E. coli* cultures and the third lanes show the sediment of induced *E. coli* cultures. Including the polyhistidine-tag, *Musa*FLS1 and *Musa*FLS3 have a mass of about 41 kDa. The *Color Prestained Protein Standard, Broad Range* (NEB) was used as a standard.

**References**

Almeida, J.R., D'Amico, E., Preuss, A., Carbone, F., de Vos, C.H., Deiml, B., et al. (2007). Characterization of major enzymes and genes involved in flavonoid and proanthocyanidin biosynthesis during fruit development in strawberry (Fragaria x ananassa). *Archives of Biochemistry and Biophysics* 465(1)**,** 61-71. doi: https://doi.org/10.1016/j.abb.2007.04.040.

Britsch, L., and Grisebach, H. (1986). Purification and Characterization of (2s)-Flavanone 3-Hydroxylase from Petunia-Hybrida. *European Journal of Biochemistry* 156(3)**,** 569-577. doi: https://doi.org/10.1111/j.1432-1033.1986.tb09616.x.

Chua, C.S., Biermann, D., Goo, K.S., and Sim, T.S. (2008). Elucidation of active site residues of Arabidopsis thaliana flavonol synthase provides a molecular platform for engineering flavonols. *Phytochemistry* 69(1)**,** 66-75. doi: 10.1016/j.phytochem.2007.07.006.

Deboo, G.B., Albertson, M.C., and Taylor, L.P. (1995). Flavanone 3-hydroyxlase transcripts and flavonol accumulation are temporally coordinate in maize anthers. *The Plant Journal* 7(5)**,** 703-713. doi: 10.1046/j.1365-313x.1995.07050703.x.

Dyballa, N., and Metzger, S. (2012). "Fast and Sensitive Coomassie Staining in Quantitative Proteomics," in *Quantitative Methods in Proteomics. Methods in Molecular Biology (Methods and Protocols),* ed. K. Marcus. (Totowa, NJ.: Humana Press).

Falcone Ferreyra, M.L., Emiliani, J., Rodriguez, E.J., Campos-Bermudez, V.A., Grotewold, E., and Casati, P. (2015). The Identification of Maize and Arabidopsis Type I FLAVONE SYNTHASEs Links Flavones with Hormones and Biotic Interactions. *Plant Physiology* 169(2)**,** 1090-1107. doi: 10.1104/pp.15.00515.

Falcone Ferreyra, M.L., Rius, S., Emiliani, J., Pourcel, L., Feller, A., Morohashi, K., et al. (2010). Cloning and characterization of a UV-B-inducible maize flavonol synthase. *The Plant Journal* 62(1)**,** 77-91. doi: 10.1111/j.1365-313X.2010.04133.x.

Gebhardt, Y., Witte, S., Forkmann, G., Lukacin, R., Matern, U., and Martens, S. (2005). Molecular evolution of flavonoid dioxygenases in the family Apiaceae. *Phytochemistry* 66(11)**,** 1273-1284. doi: 10.1016/j.phytochem.2005.03.030.

Halbwirth, H., Fischer, T.C., Schlangen, K., Rademacher, W., Schleifer, K.-J., Forkmann, G., et al. (2006). Screening for inhibitors of 2-oxoglutarate-dependent dioxygenases: Flavanone 3β-hydroxylase and flavonol synthase. *Plant Science* 171(2)**,** 194-205. doi: https://doi.org/10.1016/j.plantsci.2006.03.014.

Holton, T.A., Brugliera, F., and Tanaka, Y. (1993). Cloning and expression of flavonol synthase from *Petunia hybrida*. *The Plant Journal* 4**,** 1003-1010. doi: 10.1046/j.1365-313x.1993.04061003.x.

Kawai, Y., Ono, E., and Mizutani, M. (2014). Evolution and diversity of the 2-oxoglutarate-dependent dioxygenase superfamily in plants. *The Plant Journal* 78(2)**,** 328-343. doi: 10.1111/tpj.12479.

Kim, J.H., Lee, Y.J., Kim, B.G., Lim, Y., and Ahn, J.-H. (2008). Flavanone 3β-Hydroxylases from Rice: Key Enzymes for Favonol and Anthocyanin Biosynthesis. *Molecules and Cells* 25(2)**,** 312-316.

Kim, S., Binzel, M.L., Yoo, K.S., Park, S., and Pike, L.M. (2004). Pink (P), a new locus responsible for a pink trait in onions (Allium cepa) resulting from natural mutations of anthocyanidin synthase. *Molecular Genetics and Genomics* 272(1)**,** 18-27. doi: https://doi.org/10.1007/s00438-004-1041-5.

Laemmli, U.K. (1970). Cleavage of structural proteins during the assembly of the head of bacteriophage T4. *Nature* 227**,** 680-685. doi: 10.1038/227680a0.

Li, D.D., Ni, R., Wang, P.P., Zhang, X.S., Wang, P.Y., Zhu, T.T., et al. (2020). Molecular Basis for Chemical Evolution of Flavones to Flavonols and Anthocyanins in Land Plants. *Plant Physiology* 184(4)**,** 1731-1743. doi: 10.1104/pp.20.01185.

Martens, S., Forkmann, G., Britsch, L., Wellmann, F., Matern, U., and Lukacin, R. (2003). Divergent evolution of flavonoid 2-oxoglutarate-dependent dioxygenases in parsley. *FEBS Letters* 544(1-3)**,** 93-98. doi: 10.1016/s0014-5793(03)00479-4.

Martens, S., Forkmann, G., Matern, U., and Lukacin, R. (2001). Cloning of parsley flavone synthase I. *Phytochemistry* 58(1)**,** 43-46. doi: 10.1016/s0031-9422(01)00191-1.

Nakajima, J., Tanaka, Y., Yamazaki, M., and Saito, K. (2001). Reaction mechanism from leucoanthocyanidin to anthocyanidin 3-glucoside, a key reaction for coloring in anthocyanin biosynthesis. *Journal of Biological Chemistry* 276(28)**,** 25797-25803. doi: 10.1074/jbc.M100744200.

Owens, D.K., Alerding, A.B., Crosby, K.C., Bandara, A.B., Westwood, J.H., and Winkel, B.S. (2008). Functional analysis of a predicted flavonol synthase gene family in Arabidopsis. *Plant Physiology* 147(3)**,** 1046-1061. doi: 10.1104/pp.108.117457.

Park, S., Kim, D.H., Lee, J.Y., Ha, S.H., and Lim, S.H. (2017). Comparative Analysis of Two Flavonol Synthases from Different-Colored Onions Provides Insight into Flavonoid Biosynthesis. *Journal of Agricultural and Food Chemistry* 65(26)**,** 5287-5298. doi: 10.1021/acs.jafc.7b01036.

Park, S., Kim, D.H., Park, B.R., Lee, J.Y., and Lim, S.H. (2019). Molecular and Functional Characterization of Oryza sativa Flavonol Synthase (OsFLS), a Bifunctional Dioxygenase. *Journal of Agricultural and Food Chemistry* 67(26)**,** 7399-7409. doi: 10.1021/acs.jafc.9b02142.

Reddy, A.M., Reddy, V.S., Scheffler, B.E., Wienand, U., and Reddy, A.R. (2007). Novel transgenic rice overexpressing anthocyanidin synthase accumulates a mixture of flavonoids leading to an increased antioxidant potential. *Metabolic Engineering* 9(1)**,** 95-111. doi: https://doi.org/10.1016/j.ymben.2006.09.003.

Saito, K., Kobayashi, M., Gong, Z.Z., Tanaka, Y., and Yamazaki, M. (1999). Direct evidence for anthocyanidin synthase as a 2-oxoglutarate-dependent oxygenase: molecular cloning and functional expression of cDNA from a red forma of Perilla frutescens. *The Plant Journal* 17(2)**,** 181-189.

Shen, X., Martens, S., Chen, M., Li, D., Dong, J., and Wang, T. (2010). Cloning and characterization of a functional flavanone-3ss-hydroxylase gene from Medicago truncatula. *Molecular Biology Reports* 37(7)**,** 3283-3289. doi: https://doi.org/10.1007/s11033-009-9913-8.

Shimada, S., Inoue, Y.T., and Sakuta, M. (2005). Anthocyanidin synthase in non-anthocyanin-producing Caryophyllales species. *The Plant Journal* 44(6)**,** 950-959. doi: 10.1111/j.1365-313X.2005.02574.x.

Wellmann, F., Lukacin, R., Moriguchi, T., Britsch, L., Schiltz, E., and Matern, U. (2002). Functional expression and mutational analysis of flavonol synthase from Citrus unshiu. *European Journal of Biochemistry* 269(16)**,** 4134-4142. doi: 10.1046/j.1432-1033.2002.03108.x.

Wilmouth, R.C., Turnbull, J.J., Welford, R.W., Clifton, I.J., Prescott, A.G., and Schofield, C.J. (2002). Structure and mechanism of anthocyanidin synthase from *Arabidopsis thaliana*. *Structure* 10(1)**,** 93-103. doi: 10.1016/s0969-2126(01)00695-5.

Xu, F., Cheng, H., Cai, R., Li, L.L., Chang, J., Zhu, J., et al. (2008). Molecular cloning and function analysis of an anthocyanidin synthase gene from Ginkgo biloba, and its expression in abiotic stress responses *Molecules and Cells* 26(6)**,** 536-547.
